# Human Umbilical Cord Perivascular Cells Exhibited Enhanced Migration Capacity towards Hepatocellular Carcinoma in Comparison with Bone Marrow Mesenchymal Stromal Cells: A Role for Autocrine Motility Factor Receptor

**DOI:** 10.1155/2014/837420

**Published:** 2014-07-23

**Authors:** Juan Bayo, Esteban Fiore, Jorge B. Aquino, Mariana Malvicini, Manglio Rizzo, Estanislao Peixoto, Laura Alaniz, Flavia Piccioni, Marcela Bolontrade, Osvaldo Podhajcer, Mariana G. Garcia, Guillermo Mazzolini

**Affiliations:** ^1^Laboratorio de Terapia Génica, Facultad de Ciencias Biomédicas, Universidad Austral, B1629ODT Derqui-Pilar, Buenos Aires, Argentina; ^2^CONICET (Consejo Nacional de Investigaciones Científicas y Técnicas), C1033AAJ Buenos Aires, Argentina; ^3^Laboratorio de Terapia Molecular y Celular, Fundación Instituto Leloir, C1405BWE Buenos Aires, Argentina; ^4^Unidad de Hígado, Hospital Universitario Austral, Universidad Austral, B1629ODT Derqui-Pilar, Argentina

## Abstract

Hepatocellular carcinoma (HCC) is the third cause of cancer-related death worldwide. Unfortunately, the incidence and mortality associated with HCC are increasing. Therefore, new therapeutic strategies are urgently needed and the use of mesenchymal stromal cells (MSCs) as carrier of therapeutic genes is emerging as a promising option. Different sources of MSCs are being studied for cell therapy and bone marrow-derived cells are the most extensively explored; however, birth associated-tissues represent a very promising source. The aim of this work was to compare the *in vitro* and *in vivo* migration capacity between bone marrow MSCs (BM-MSCs) and human umbilical cord perivascular cells (HUCPVCs) towards HCC. We observed that HUCPVCs presented higher *in vitro* and *in vivo* migration towards factors released by HCC. The expression of autocrine motility factor (AMF) receptor, genes related with the availability of the receptor on the cell surface (caveolin-1 and -2) and metalloproteinase 3, induced by the receptor activation and important for cell migration, was increased in HUCPVCs. The chemotactic response towards recombinant AMF was increased in HUCPVCs compared to BM-MSCs, and its inhibition in the conditioned medium from HCC induced higher decrease in HUCPVC migration than in BM-MSC. Our results indicate that HUCPVCs could be a useful cellular source to deliver therapeutic genes to HCC.

## 1. Introduction

Hepatocellular carcinoma (HCC) is one of the leading causes of cancer-related death worldwide. At early stages of the disease only a small fraction of patients are amenable to curative treatments that include surgical resection, liver transplantation, or radiofrequency ablation [1]. For patients with advanced HCC, the multikinase inhibitor sorafenib shows limited survival benefits in comparison with placebo [2]. Thus, the development of new therapeutic approaches is critical, and cellular therapy emerges as a new potential strategy to deliver therapeutic genes to HCC.

Mesenchymal stromal cells (MSCs) constitute a heterogeneous population of cells that include adult multipotent cells [3]. MSCs are present in adult tissues and their involvement in repair mechanisms has been demonstrated as the result of their ability to migrate towards sites of injury, their capacity to differentiate into tissues of mesodermal origin (adipocytes, osteoblasts, and chondroblasts), and their immunoregulatory properties [4]. Moreover, the possibility of easily expanding these cells* in vitro *makes them useful tools for therapeutic use in regenerative medicine, immunomodulation purposes and also as cellular carriers for therapeutic genes [5].

MSCs can be isolated from different tissues. Among them, bone marrow- (BM-) derived stromal cells were the first described and are the most frequently used [6]. However, the requirement of invasive methods to obtain BM-MSCs is in support of using alternative sources such as adipose tissue, peripheral blood, and dental pulp [7]. In addition to these adult tissues, MSCs can be obtained from birth-associated tissues including placenta, amnion, and umbilical cord with the advantage of their availability avoiding the need of invasive procedures and eliminating other ethical concerns. Different types of MSCs have been obtained from the umbilical cord depending on if they were isolated from the whole umbilical cord (UC-MSCs), Wharton's Jelly (WJ-MSCs), the umbilical cord blood (CB-MSCs) [8], or perivascular areas (human umbilical cord perivascular cells, HUCPVCs) [9]. In particular, HUCPVCs may be advantageous candidates for cell therapy due to their lower donor variability, faster doubling time, and ready availability [10].

It has been demonstrated that MSCs from different sources display similar phenotypic and cytological characteristics [11–13]; however, differences in their functional role have also been previously described. For instance, BM and WJ-derived MSCs have different proliferative capacity [11] and secretome and proteomic profiles [14, 15].

The establishment and spread of a tumor is a process that involves a complex cross-talk between cancer cells and the tumor microenvironment. HCC cells were found to be modulated by the tumor milieu through the secretion of several factors, and the tumor cells modify their microenvironment in order to promote their own growth [16]. Particularly, MSCs express receptors for various cytokines and chemokines allowing them to migrate towards HCC tumors [17]. Thus, recruitment of MSCs to cancer microenvironment is likely mediated by the interaction of cytokines/chemokines and their specific receptors. As an example, it has been demonstrated that CXCR1 is involved in UC-MSC migration towards glioma [18] and that overexpression of CXCR1 [19] or CXCR4 in these cells [20] increases their homing into tumors. In addition, MSCs were engineered to express cytotoxic cytokines for treatment of lung tumors and non-Hodgkin's lymphomas [21, 22]. Thus, the possibility of increasing MSC migratory capacity and engraftment into tumors could enhance beneficial effects of therapeutic genes.

The aim of this work was to compare HUCPVCs and the “gold standard” BM-MSCs migratory capacity towards factors released from HCC and to analyze mechanisms therein involved.

## 2. Materials and Methods

### 2.1. Cell Lines

Human HCC cell line HuH7 was kindly provided by Professor Jesus Prieto (CIMA, University of Navarra, Pamplona, Spain) [23]. LX-2 cell line (human hepatic stellate cells generated by spontaneous immortalization in low serum conditions) was kindly provided by Dr. Scott Friedman (Division of Liver Diseases, Mount Sinai School of Medicine, New York, NY, USA) [24]. Human microvascular endothelial cells (HMEC-1) were from CDC (Centers for Disease Control, Atlanta, GA, USA) and WI-38 (human fibroblast cell line) from the American Type Culture Collection. Cell lines were cultured in complete DMEM (2 *μ*M glutamine, 100 U/mL penicillin, 100 mg/mL streptomycin) and 10% heat-inactivated fetal bovine serum (FBS). Primary culture of HCC cells (HC-PT-5) was previously generated in our laboratory [25]. The collection of the sample and the project was approved by the Institutional Evaluation Committee (CIE) from School of Biomedical Sciences, Austral University (Protocol no. 11-007), and written informed consent was obtained from the patient. HC-PT-5 was cultured up to 8 passages in 70% DMEM/30% F12 (Invitrogen/Life Technologies) culture medium supplemented with 2 *μ*M glutamine, 100 U/mL penicillin, 100 mg/mL streptomycin, and 10% FBS.

### 2.2. Isolation of BM-MSCs and HUCPVCs

BM-MSCs were obtained from healthy donors (Hospital Naval Pedro Mallo, Buenos Aires, Argentina) as described previously [25]. HUCPVCs were isolated from umbilical cord obtained from healthy donors at the Hospital Universitario Austral (Pilar, Buenos Aires, Argentina) using a protocol adapted from Sarugaser et al. [9]. In brief, umbilical cords were dissected and vessels with their surrounding Warthon's Jelly were pulled out. The perivascular mesenchymal tissue was removed from the vessels and mechanically disrupted. Minced fragments were plated in complete DMEM low glucose/20% FBS (Internegocios S.A., Argentina). After 7-day incubation, nonadherent cells and minced fragments were removed and adherent HUCPVCs were cultured and used for different experiments at passages 4 to 6.

MSCs were characterized according to the International Society for Cellular Therapy (ISCT) guidelines [26]. Phenotype characteristics of MSCs were determined by flow cytometry with anti-human PE conjugated antibodies against CD14, CD34, CD44, CD73, CD90, and CD105 (BD Biosciences) for 30 min. Samples were analyzed using a FACSCalibur flow cytometer (Becton Dickinson), and data acquired were analyzed using Cyflogic software (CyFlo Ltd.).

### 2.3. Conditioned Medium

To obtain tumor conditioned medium (TCM), HuH7 cells (2 × 10^6^) or HC-PT-5 cells (5 × 10^6^) were inoculated subcutaneously (s.c.) into the right flank of nude mice. When tumors reached 200 mm^3^ in size approximately, tumors were dissected and minced into pieces smaller than 1 mm^3^ and transferred to a 24-well tissue culture plate (6 fragments/well) with 500 *μ*L of complete DMEM without FBS. Cell conditioned medium (CCM) was obtained from cell lines cultured as described above. Then, cells that reached a 90% of confluence were washed with PBS and cultured with complete DMEM without FBS. In both cases, 18 hours later, conditioned media were harvested and stored at −80°C until use.

### 2.4. *In Vitro* Migration Assays

*In vitro* migration was performed using a 48-Transwell microchemotaxis Boyden Chamber unit (Neuroprobe, Inc.) as previously described [25]. MSCs (1.2 × 10^3^ cells/well) were placed in the upper chamber and DMEM, CCM, TCM, or rAMF were applied to the lower chamber of the transwell unit. For blocking experiments, TCM were preincubated for 60 min with anti-AMF antibody or isotype control IgG. All systems were left at 37°C in a 5% CO_2_ humidified atmosphere for 4 hours except for experiments involving rAMF that were maintained for 18 hours. Cells attached to the lower side of the membrane were fixed in 2% formaldehyde, stained with 4′,6-diamidino-2-phenylindole dihydrochloride (DAPI, Sigma-Aldrich), and counted using fluorescent-field microscopy at 100x. Captured images from three representative visual fields were analyzed using Cell Profiler software (http://www.cellprofiler.com/) and the mean number of cells/field ± SEM was calculated.

### 2.5. Cell Adhesion Assays

For analyses of MSC adhesion to endothelial cells, 2 × 10^5^ HMEC-1 were seeded in 96-well microplates and cultured for 1 day prior to the assay. Coated wells were incubated for 5 minutes with 0.1 mL of 5 × 10^4^ cells/mL of Fast-DiO prelabelled MSCs. Cell suspension was discarded and attached cells were fixed with 2% paraformaldehyde. Cells were counted using fluorescent-field microscopy at 200x: pictures taken from ten representative visual fields were analyzed using Cell Profiler software (http://www.cellprofiler.com/) and the mean number of cells/field ± SEM was calculated.

### 2.6. Reverse Transcription-Polymerase Chain Reaction (RT-PCR)

Total RNA of MSCs was extracted using Trizol Reagent (Sigma-Aldrich Co., St. Louis, MO). Total RNA (4 *μ*g) was reverse-transcribed with 200 U of SuperScript II Reverse Transcriptase (Invitrogen, Carlsbad, CA) using 500 ng of Oligo (dT) primers. cDNAs were subjected to real-time polymerase chain reaction (qPCR) (Stratagene Mx3005p, Stratagene, La Jolla, CA, USA). For qRT-PCR, the mRNA levels of CXCR1, CXCR2, CCR2, IL-6 receptor (IL-6R), IL-6 signal transducer (IL-6ST), AMF receptor (AMFR), metalloproteinase 3 (MMP3), insulin-like growth factor-binding protein 3 (IGFBP3), caveolin-1 (CAV-1), and caveolin-2 (CAV-2) were quantified by SYBR Green (Invitrogen), using the following primers: CXCR1 forward 5′-TTTTCCGCCAGGCTTACCAT-3′ and reverse 5′-AACACCATCCGCCATTTTGC-3′; CXCR2 forward 5′-TAAGTGGAGCCCCGTGGGG-3′ and reverse 5′-TGGGCTCAGGGGCAGGATG-3′; CCR2 forward 5′-CGAGAGCGGTGAAGAAGTCA-3′ and reverse 5′-AGCATGTTGCCCACAAAACC-3′; IL-6R forward 5′-GCACTTGCTGGTGGATGTTC-3′ and reverse 5′-AGCCTTTGTCGTCAGGGATG-3′; IL-6ST forward 5′-CCCACCTCATGCACTGTTGA-3′ and reverse 5′-TTATGTGGCGGATTCGGCTT-3′; AMFR forward 5′-ACAAGATGTGGGCCTTGCAAGA-3′ and reverse 5′-AAAACGCAGTGCTCCCAGGATA-3′; MMP3 forward 5′-ACGCCAGCCAACTGTGATCCT-3′ and reverse 5′-ATATGCGGCATCCACGCCTGAA-3′; IGFBP3 forward 5′-ACTGTGGCCATGACTGAG-3′ and reverse 5′-AGAGTCTCCCTGAGCCTGA-3′; CAV-1 forward 5′-AATCCAAGCATCCCTTTGCCCA-3′ and reverse 5′-ACCAGGCAGCTTTCTGTACGA-3′; CAV-2 forward 5′-GAGAGACAGGGGAGTTGTCAACTT-3′ and reverse 5′-GCCCGGCCCAGAAATAATGAGAT-3′. PCR amplifications were carried out using a cycle of 95°C for 10 minutes and 45 cycles under the following parameters: 95°C for 30 seconds, 58°C for 60 seconds, 72°C for 30 seconds. At the end of PCR reaction, the temperature was increased from 60°C to 95°C at a rate of 2°C/min, and the fluorescence was measured every 15 seconds to construct the melting curve. Values were normalized to levels of glyceraldehyde-3-phosphate dehydrogenase (GAPDH; used as housekeeping) transcript (forward 5′-CATCTCTGCCCCCTCTGCTG-3′ and reverse 5′-GCCTGCTTCACCACCTTCTTG-3′). Data were processed by the ΔΔCt method. The relative amount of the PCR product amplified from BM-MSCs was set as 1. A nontemplate control (NTC) was run in every assay, and all determinations were performed as triplicates in three separated experiments.

### 2.7. Mice and* In Vivo* Experiments

Six- to eight-week-old male nude mice (Nu/Nu) were purchased from CNEA (Comisión Nacional de Energía Atómica, Ezeiza, Buenos Aires, Argentina). Subcutaneous HuH7 tumors (2 × 10^6^cells) were established and 10 days later BM-MSCs or HUCPVCs were intravenously (i.v.) injected. Tumor growth was assessed by calliper measurement, and tumor volume (mm^3^) was calculated by the formula *π*/6 × larger diameter × (smaller diameter)^2^. For* in vivo* migration studies, MSCs (5 × 10^5^) were prelabeled with CM-DiI for histological analysis and DiR (Molecular Probes, Invitrogen) for fluorescence imaging (FI). FI was performed using the Xenogen* In Vivo* Imaging System (IVIS; Caliper Life Sciences, Hopkinton, MA, USA) 1 hour after MSC injection and every day until experimental end point. At day 3 mice were sacrificed and isolated tissues were exposed to FI. Images represent the radiant efficiency and were analyzed with IVIS Living Image (Caliper Life Sciences) software. Regions of interest (ROI) were automatically drawn around the isolated organs to assess the fluorescence signal emitted. For the total signal present in mice, results were expressed as total radiant efficiency in units of photons/second within the region of interest [p/s]/[*μ*W/cm^2^]. Signal present in tumor, liver, spleen, or lungs was expressed as percentage of total signal.

### 2.8. Detection of BM-MSCs by Fluorescence

To detect CM-DiI+ cells within tumors, frozen sections were mounted in mounting media with DAPI (Vector Laboratories, Inc.) and observed under a fluorescence microscope at 200x.

### 2.9. Statistical Analyses

Unpaired 2-sided Student's *t*-test and one-way analysis of variance followed by posttests or Kruskal-Wallis and Dunn's posttests (GraphPad Prism Software) were used for statistical analyses. *P* values lower than 0.05 were considered as statistically significant.

## 3. Results

### 3.1. Characterization of BM-MSCs and HUCPVCs

**Figure 1:**
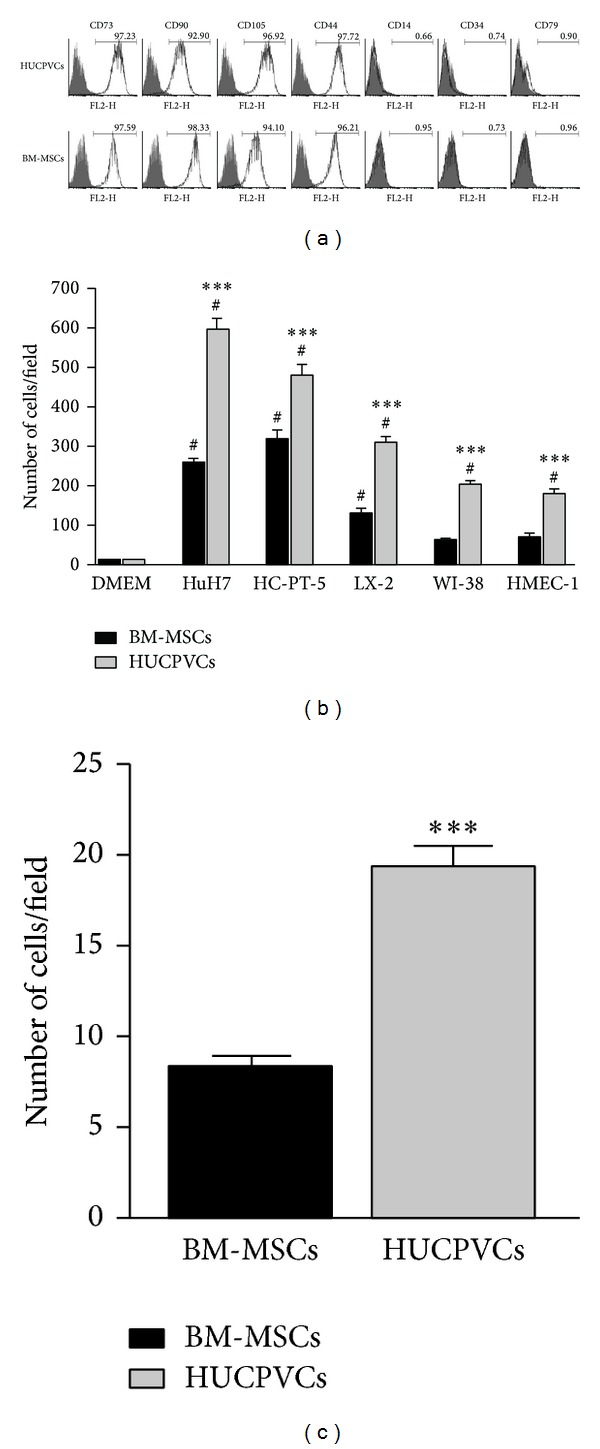
Characterization of BM-MSCs and HUCPVCs. (a) Flow cytometry analysis of cell surface markers of both types of MSCs. Grey area indicates background fluorescence with IgG isotype control. One representative experiment is shown. (b)* In vitro* migration of BM-MSCs (black bars) or HUCPVCs (grey bars) towards CCM from HCC (HuH7 and HC-PT-5), hepatic stellate cells (LX-2), fibroblasts (WI-38), or endothelial cells (HMEC-1). Bars represent the average of MSCs/field (10x) ± SEM from three representative visual fields. Results are representative of 3 independent experiments. ^#^ *P* < 0.001 versus DMEM; ***P < 0.001 versus BM-MSCs. (c) Adhesion towards endothelial cells of BM-MSCs (black bars) or HUCPVCs (grey bars). Results are representative of 3 independent experiments. ***P < 0.001 versus BM-MSCs.

In accordance with the criteria for defining MSCs of the International Society for Cellular Therapy (ISCT) [26], surface marker expression of BM-MSCs and HUCPVCs was evaluated by flow cytometry. Both types of MSCs showed similar phenotypic characteristics and were found to express CD73, CD90, CD105, and CD44 but not to express the hematopoietic markers CD14, CD34, or CD79 (Figure 1(a)). We next decided to evaluate the* in vitro* migration capacity of MSCs towards cell culture conditioned media (CCM) obtained from HCC cell lines (HuH7 and HC-PT-5), hepatic stellate cells (LX-2), fibroblasts (WI-38), and endothelial cells (HMEC-1). Interestingly, a higher migratory capacity towards all the CCM was found for HUCPVCs when compared to BM-MSCs (Figure 1(b)). Moreover, in contrast to our previous results observed with BM-MCSs [25], HUCPVCs showed capability to migrate to CCM derived from nontumoral components (fibroblast and endothelial cells). Besides their capacity to migrate toward factors secreted by HCC, the arrest of MSCs within the microvasculature is considered a critical step for an efficient homing and anchorage to tumors. Therefore, we next decided to evaluate adhesion ability of MSCs and observed that HUCPVCs showed an increased* in vitro *adhesion to HMEC-1 endothelial cells in comparison with BM-MSCs (Figure 1(c)).

### 3.2. *In Vivo* Migration of BM-MSCs and HUCPVCs towards HCC

**Figure 2:**
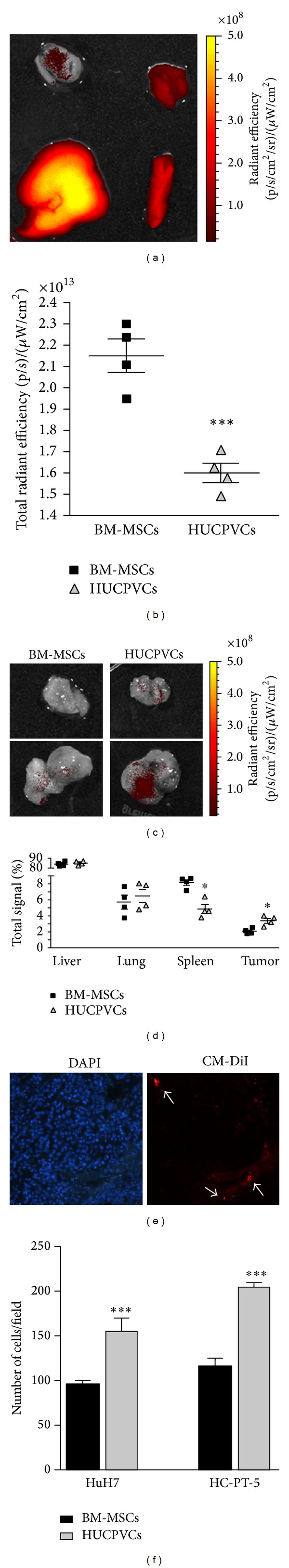
*In vivo* migration of BM-MSCs and HUCPVCs. CM-DiI and DiR prelabeled MSCs were i.v. injected in s.c. HuH7 tumor-bearing mice. At day 3 mice were sacrificed and organs were removed; lungs, livers, spleen (a) and tumors (c) were exposed to obtain FI. Images represent the radiant efficiency. Representative images are shown. (b) Total FI for injected BM-MSCs or HUCPVCs was calculated by measuring the region of interest (ROI) for all the tissues isolated and results were expressed as total radiant efficiency [p/s]/[*μ*W/cm^2^]. ***P < 0.001. (d) Signal present in the isolated liver, spleen, lungs and tumors was represented as percentage of total signal for BM-MSCs or HUCPVCs-injected mice. *P < 0.05 versus BM-MSCs. (e) Microscopic analysis of transplanted CM-DiI-labeled MSCs (red signal indicated by arrows) and DAPI staining in frozen sections of tumors. ×200 magnification. (f)* In vitro* migration of MSCs to TCM derived from HuH7 or HC-PT-5 s.c. tumors. Bars represent the average of MSCs/field (10x) ± SEM from three representative visual fields. Results are representative of 3 independent experiments. ***P < 0.001 versus BM-MSCs.

To further characterize MSC behavior* in vivo*, noninvasive migration assay was performed. CM-DiI and DiR prelabelled BM-MSCs or HUCPVCs were i.v. injected in HCC tumor-bearing mice in order to evaluate MSC recruitment. Similar to our previous observation with BM-MSCs [25], at 3 days after cell transplantation a positive signal corresponding to HUCPVCs was found in liver, lungs, spleen, and s.c. tumors (Figure 2(a)). Despite the fact that total signal was lower in mice injected with HUCPVCs compared to those injected with BM-MSCs (Figure 2(b)), the percentage of total signal corresponding to s.c. tumor locations was increased in mice administered with HUCPVCs in comparison with animals that received BM-MSCs (Figures 2(c) and 2(d)), indicating an enhanced engraftment of HUCPVCs into HCC tumors. In the other evaluated tissues, signal intensity was similar for BM-MSC or HUCPVCs in lung and liver and it was comparatively reduced in the spleen of HUCPVCs-injected mice (Figure 2(d)). Presence of MSCs in the s.c. tumors was also confirmed by fluorescence microscopy (Figure 2(e)). Finally, we evaluated whether MSCs might present differential migratory capacity towards CM obtained from s.c. tumors (TCM). Interestingly, a greater* in vitro* migratory capacity towards TCM from HCC was observed for HUCPVCs when compared to BM-MSCs (Figure 2(f)).

### 3.3. AMFR Was Highly Expressed in HUCPVCs

**Figure 3:**
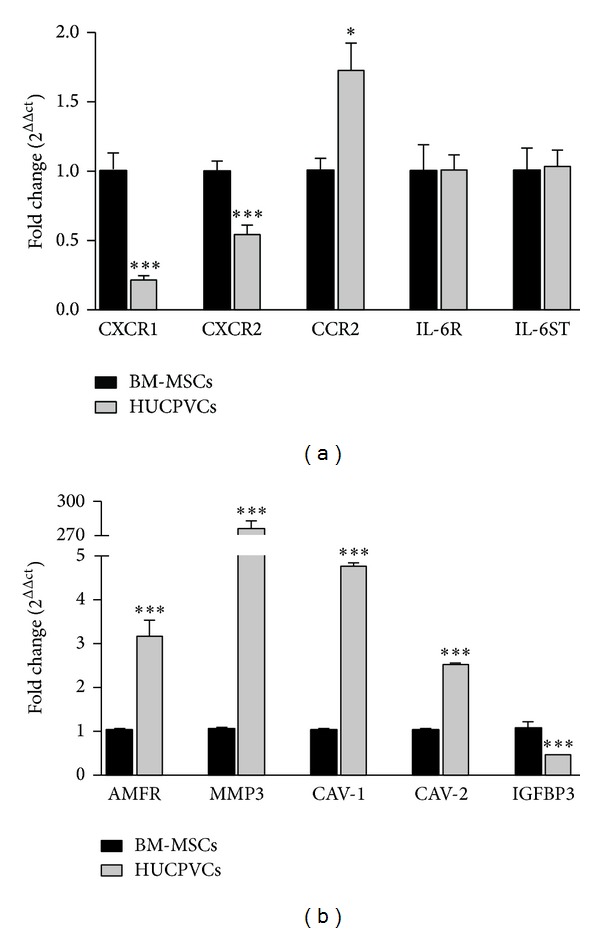
Differential expression of cytokines/chemokines receptors and AMF/AMFR pathway in MSCs. Expression of cytokines and chemokines receptors (a) and AMF/AMFR axis proteins (b) was evaluated in BM-MSCs (black bars) or HUCPVCs (grey bars) by qPCR. ***P < 0.001 versus BM-MSCs.

In order to evaluate mechanisms partially explaining the differential migratory capacity of HUCPVCs compared to BM-MSCs towards tumor released factors, we analyzed the expression of some chemokine's receptor likely involved in MSC recruitment towards HCC. Taking into account several reports demonstrating that interleukin- (IL-) 8, GRO, chemokine (C-C motif) ligand (CCL)-2, and IL-6 are among the most relevant factors in HCC [17], we decided to evaluate by qPCR the expression of CXCR1, CXCR2, CCR2, IL-6R, and IL-6ST. Interestingly, constitutive CXCR1 and CXCR2 mRNA expression was found to be lower and CCR2 slightly higher in HUCPVCs when compared to BM-MSCs, while IL-6R and IL-6ST expression was similar in both MSCs sources (Figure 3(a)). Next, we decided to evaluate the axis of the autocrine motility factor (AMF), a cytokine with chemotactic effect on MSCs as recently observed by our group [27]. By qPCR, a significantly higher expression of the AMF receptor (AMFR) was found in HUCPVCs when compared to BM-MSCs. Similarly, genes known to be related to the availability of the receptor in the cell surface such as caveolin-1 (CAV-1) and caveolin-2 (CAV-2) were also highly expressed in HUCPVCs as well as the metalloproteinase 3 (MMP3), necessary to the transmigration process. In contrast, expression levels of insulin-like growth factor-binding protein 3 (IGFBP3), a protein that negatively regulates AMF/AMFR pathway, were found to be reduced in HUCPVCs when compared to BM-MSCs (Figure 3(b)).

### 3.4. HUCPVCs Displayed Enhanced Migration towards AMF* In Vitro*

**Figure 4:**
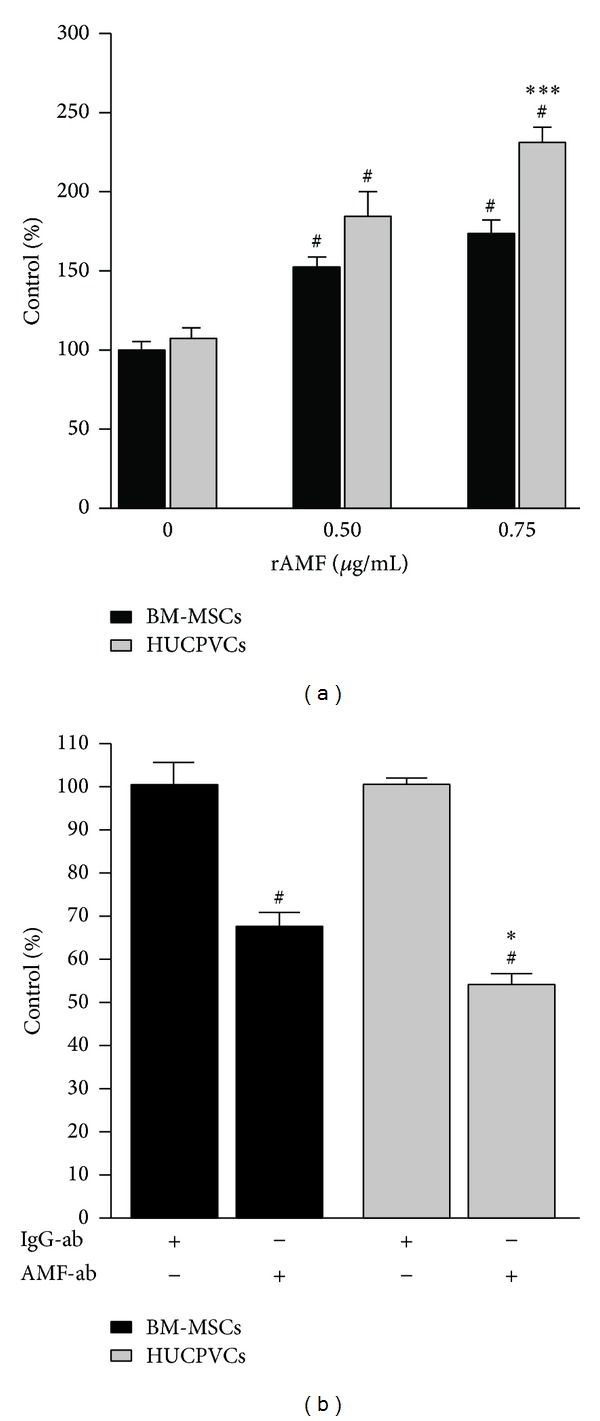
HUCPVCs showed enhanced migration towards AMF in comparison with BM-MSCs. (a)* In vitro* migration of BM-MSCs (black bars) or HUCPVCs (grey bars) towards rAMF. ^#^ *P* < 0.05 versus DMEM (0 *μ*g/mL rAMF); *P < 0.05 versus BM-MSCs. (b)* In vitro* migration of BM-MSCs (black bars) or HUCPVCs (grey bars) towards HC-PT-5 TCM preincubated with anti-AMF antibody (AMF-ab) or control isotype (IgG-ab) was evaluated. ^#^ *P* < 0.05 versus IgG-ab; *P < 0.05 versus BM-MSCs. Bars represent the average of MSCs/field (10x) ± SEM from three representative visual fields. Results are representative of 3 independent experiments.

We have previously demonstrated that AMF, a cytokine produced by HCC cells, plays a critical role in MSC migration [27]. Due to the increased AMFR and AMF-AMFR-related proteins expression in HUCPVCs, we decided to test the* in vitro* migration response to the recombinant AMF (rAMF) of both types of MSCs using a chemotaxis assay. A significantly higher migration to different doses of rAMF (0.5 and 0.75 *μ*g/mL) was observed for HUCPVCs when compared to BM-MSCs (Figure 4(a)). In spite of different types of MSCs showing similar reduction in migration levels (50% of control) towards HuH7 TCM after the blockage with anti-AMF antibody (data not shown), preincubation of HC-PT-5 TCM with anti-AMF antibody (AMF-ab) resulted in a further reduction in HUCPVCs migration capacity (54% of control) when compared to BM-MSCs (67% of control) (Figure 4(b)).

## 4. Discussion

The aim of this study was to assess the capability of different sources of MSCs to migrate towards HCC released factors in order to select those showing higher tumor recruitment capacity for future therapeutic applications. Our results indicated that although both types of MSCs share similar phenotypic characteristics as MSCs, HUCPVCs have a higher potential to migrate* in vitro* towards the conditioned medium derived not only from HCC cells, but also from nontumoral components of the tumor microenvironment like activated hepatic stellate cells (LX-2), fibroblasts (WI-38), or tumor microendothelial cells (HMEC-1). Moreover, HUCPVCs also showed an enhanced adhesion capacity to HMEC-1 cells compared to BM-MSCs. This enhanced adhesion to endothelial cells could allow a more efficient arrest in tumor microvasculature which is a required step for MSC recruitment into HCC. In line with this, we observed that HUCPVCs also exhibited greater migration capacity* in vivo *towards experimental HCC. In contrast to that observed in the* in vitro* migration experiments, HUCPVCs showed a similar migration capability towards nontumoral tissues like liver or lung and also showed a lower recruitment to the spleen. Although HUCPVCs migrated better than BM-MSCs toward activated hepatic stellate cells* in vitro*, no differences were observed in* in vivo* migration to normal liver, where hepatic stellate cells are quiescent. In addition, it should be noted that mice injected with HUCPVCs showed a lower total signal than those injected with BM-MSCs, probably due to differences in the DiR uptake for both kinds of cells or in the number of inoculated cells. These results are in agreement with previous works showing that MCSs derived from umbilical cord are able to migrate towards experimental tumors such as glioma [28], breast cancer [29], and lung adenocarcinoma [30]. Nonetheless, to the best of our knowledge, this is the first report describing the comparative migration capacity of human umbilical cord perivascular cells towards human HCC.

Tumor-homing is thought to be mediated by interactions of cytokines/chemokines with their specific receptors. A number of cytokines, growth factors, and chemokines are secreted by HCC cells and their microenvironment [17]. We have recently found that AMF is critical for MSC migration toward HCC* in vitro* and* in vivo*. Furthermore, other factors released by tumor stroma components like IL-8, GRO, IL-6, or CCL-2 could also be involved in MSC recruitment to HCC. Thus, differences observed between HUCPVCs and BM-MSCs in their* in vitro* and* in vivo* migration capacity towards HCC seem at least partially related to differences in the expression of cytokine/chemokine receptors. Some studies have screened for similarities or differences among BM-MSCs and UC-MSCs at the molecular level [31, 32]. In addition, a previous report demonstrated that migration of UCB-MSC towards glioma cells was higher when compared to BM-MSCs likely due to increased levels of the IL-8 receptor, CXCR1, and CXCR2 in the former cell type [18]. In contrast to these observations, we found that HUCPVCs presented lower levels of CXCR1 and CXCR2 expression compared to BM-MSCs and these differences could be explained partially due to differences in tissue source and culture conditions and merit further investigation. The analysis of IL-6R and IL6-ST expression showed similar levels in both types of MSCs, while levels of MCP-1 receptor (CCR2) were slightly increased in HUCPVCs. Interestingly, we found that not only AMFR but also CAV-1 and CAV-2, which are genes that regulate the availability of AMFR on cell surface [33], were overexpressed in HUCPVCs compared to BM-MSCs. In line with this, a reduced expression level of IGFBP3, a protein which can bind to AMF blocking its binding to AMFR [34], was found in HUCPVCs when compared to BM-MSCs. Moreover, an increased expression of MMP3 was observed in HUCPVCs. This matrix metalloproteinase is involved in AMF-induced migration and it is necessary for cell invasion and proteolysis of the extracellular matrix [35]. Finally, and consistent with an increase in the expression of genes related to the AMF/AMFR pathway, not only HUCPVCs show an enhanced* in vitro* migration towards rAMF, but also the specific blockage of AMF in HC-PT-5 TCM was able to reduce HUCPVCs migration to a greater level when compared to BM-MSCs.

The potential use of MSCs in cancer treatment as carriers of therapeutic genes has raised some concerns about the safety of their use in the clinic. There are some studies that indicated that MSCs have protumorigenic capacity due to their immunosuppression properties, modulation of the epithelial-to-mesenchymal transition, and induction of angiogenesis [36]. However, others have demonstrated that MSCs decreased HCC growth [37, 38]. Similarly, we have previously demonstrated that BM-MSCs did not modify tumor growth* in vivo* [25]. Moreover, we observed that HUCPVCs did not induce tumor growth when injected i.v. in Huh7 s.c. tumor-bearing mice (data not shown). The use of HUCPVCs as cellular carriers could also have the advantage of its allogeneic application because of its low immunogenicity [9]. In this regard, HUCPVCs could be useful as carriers of therapeutic genes for cancer patients, where isolation of BM or adipose tissue-derived MSCs could be less responsive to chemotactic factors, and might display higher immunosuppression capacities than MSCs from healthy donors [39] or even present genetic abnormalities.

## 5. Conclusions

In summary, our results demonstrate a greater migration capacity to HCC of HUCPVCs when compared to BM-MSCs not only* in vitro *but also* in vivo, *likely due to an increase in migratory response to AMF and to an enhanced adhesive capacity to tumor microvasculature. Considering their availability and that no invasive procedures are required to obtain HUCPVCs, these cells have advantages over BM-MSCs as candidates for carriers of therapeutic genes for the treatment of HCC.
